# Thrombocytopenia in Sepsis

**DOI:** 10.3390/life15020274

**Published:** 2025-02-11

**Authors:** Alireza Setarehaseman, Abbas Mohammadi, Robert W. Maitta

**Affiliations:** 1 University Hospitals Cleveland Medical Center, Case Western Reserve University School of Medicine, Cleveland, OH 44106, USA; alireza.setarehaseman@uhhospitals.org; 2 Department of Internal Medicine, Valley Health System, Las Vegas, NV 89119, USA; abbas_pr2005@hotmail.com

**Keywords:** platelets, sepsis, thrombocytopenia, infection, activation, phagocytosis, leukocyte, immune

## Abstract

Platelets, traditionally known for their role in hemostasis, have emerged as key players in immune response and inflammation. Sepsis, a life-threatening condition characterized by systemic inflammation, often presents with thrombocytopenia, which at times, can be significant. Platelets contribute to the inflammatory response by interacting with leukocytes, endothelial cells, and the innate immune system. However, excessive platelet activation and consumption can lead to thrombocytopenia and exacerbate the severity of sepsis. Understanding the multifaceted roles of platelets in sepsis is crucial for developing effective therapeutic strategies. Targeting platelet-mediated inflammatory responses and promoting platelet production may offer potential avenues for improving outcomes in septic patients with thrombocytopenia. Future research should focus on elucidating the mechanisms underlying platelet dysfunction in sepsis and exploring novel therapeutic approaches to optimize platelet function and mitigate inflammation. This review explores the intricate relationship between platelets, inflammation, and thrombosis in the context of sepsis.

## 1. Introduction

Platelets are small (2–5 µm) anucleated cellular elements essential to the maintenance of coagulation homeostasis that survive in circulation for up to 10 days following release from the bone marrow in their immature forms, which then derive into smaller functional mature platelets in the vasculature [1,2]. Notably, platelets are also derived from megakaryocytes found in the lungs, which may account for about half of all platelets found in peripheral circulation, with the capacity to further reconstitute platelet counts when significant thrombocytopenia develops [3,4]. Platelet functions can be impaired under innate or acquired conditions that affect the ability of platelets to properly maintain homeostasis or to assist physiologic non-hemostatic processes [5].

In the setting of tissue injury, platelets, through a number of its receptors, adhere to the exposed subendothelial extracellular matrix at the site of injury, specifically to collagen, von Willebrand factor, laminin, fibronectin, and thrombospondin, triggering multiple signaling cascades that strengthen platelet adherence [1,6]. Further enhancement of platelet aggregation and recruitment to the injury site occurs through platelet–platelet interactions mediated by the integrin receptor αIIbβ3, which initiates the formation of a fibrin-rich hemostatic plug [1,6], as well as FcγRIIa, among other receptors, which incidentally is also important during active bacterial clearance [7].

Data from animal studies, such as murine models of sepsis induced by cecal ligation and puncture or lipopolysaccharide (LPS) administration, are crucial for dissecting the molecular mechanisms underlying platelet activation, consumption, and interactions with other immune cells [8,9,10]. These models have allowed for controlled manipulation of variables while studying platelet-specific gene knockouts or interventions to establish the physiological roles of platelets during infection. Human clinical trials, on the other hand, are essential for translating the findings from preclinical studies to the clinical setting [11,12,13]. These trials can range from observational studies examining the correlation between platelet parameters and sepsis severity to interventional studies evaluating the efficacy of novel therapies targeting platelet dysfunction or deficiencies. Advanced techniques, such as flow cytometry, electron microscopy, and single-cell RNA sequencing, are increasingly being employed to characterize platelet activation state, interactions with pathogens, and changes in platelet transcriptome and proteome in both animal models and human samples [14,15,16,17]. These approaches offer unprecedented insights into the complex role of platelets in sepsis.

This review aims to elucidate the multifaceted roles of platelets beyond hemostasis, specifically focusing on their critical contributions to the immune response, particularly in the context of sepsis. This review will explore the mechanisms by which platelets interact with pathogens and other immune cells, including their involvement in pathogen clearance, inflammation modulation, and the interplay between thrombosis and immunity. Furthermore, it will analyze the clinical implications of thrombocytopenia in sepsis, examining its prognostic value and the challenges associated with its management. Finally, this review will critically evaluate current and emerging therapeutic strategies for sepsis-associated thrombocytopenia (SAT), platelet transfusions, intravenous immunoglobulins, recombinant thrombopoietic growth factors, and targeted inhibition of key platelet mediators needed for platelet adhesion and function, highlighting their potential for improving patient outcomes.

## 2. Additional Platelet Physiological Roles

**Figure 1:**
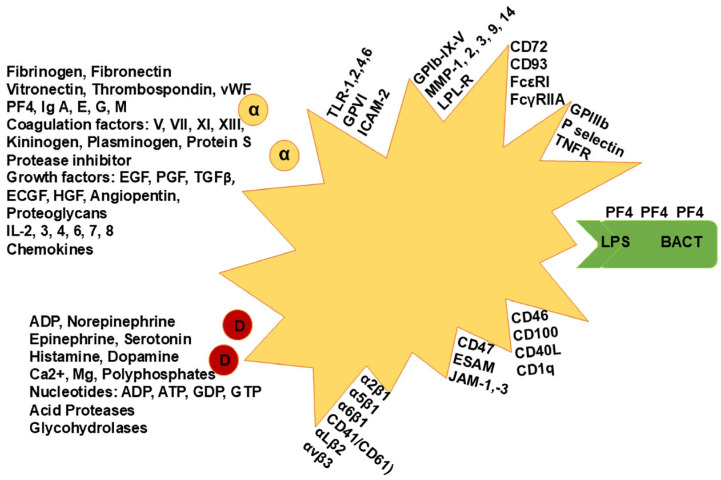
Diagram of a platelet with mediators that have roles and/or influence other cells or systems. These include chemokines, cytokines, growth factors, clotting factors, receptors, opsonins, transmitters, immunoglobulins, and enzyme inhibitors, among others. The diagram is not exhaustive and exemplifies the hundreds of mediators produced or expressed by platelets to exert their functions. Yellow circles represent α-granules and red circles represent dense granules, while green shape represents a bacterium interacting with a platelet.

The use of platelet transfusions to relieve thrombocytopenia or to address platelet dysfunction is one the most common interventions occurring in clinical practice across medical disciplines. However, the effect of platelet transfusions go far beyond coagulation, demonstrating their importance to immunity, inflammatory processes, neoplastic angiogenesis, and metastasis [18]. Platelets are capable of modulating immune responses, either through their interaction with endothelial cells and leukocytes [19,20] or through the release of antimicrobial mediators stored within platelets in response to infection [21,22]. Indeed, it has been reported that platelets in their granules contain more than 300 bioactive proteins that span functions in immunity, inflammation, cell growth, and proliferation, to name a few [3,23]. Furthermore, it has become increasingly evident that there is an interplay between the immune system, inflammation and cancer, and that platelets play a major role in their convergence [24]. Along these lines, platelets’ α-granules contain fibrinogen, von Willebrand factor (vWF), platelet factor (PF)-4, coagulation factors, immunoglobulins, growth factors, and protease inhibitors, while dense-granules are rich in adenosine diphosphate, serotonin, nucleotides, histamine, and dopamine, among others (Figure 1) [3,23]. These are some examples of mediators found in platelets that carry out functions in different physiologic responses through the body.

Platelets can be seen as the “swiss-army knife” in circulation. Through its conserved role in immune defense, pathological conditions such as heparin-induced thrombocytopenia (HIT) serve to illustrate how the intricate interplay between their activation, immune mechanisms/dysfunction, and clinical complications can disrupt normal homeostasis. HIT is a known complication of using heparin as an anticoagulant caused by the development of anti-PF4-heparin antibodies. The formation of such antibodies to PF4-heparin complexes as seen in HIT may be indicative of an evolutionarily conserved immune defense mechanism. PF4 found in platelets’ α-granules is actively released during platelet activation, binding directly to polyanions on bacteria, leading to antibody formation with specificity for PF4–polyanion complexes that target bacteria, opsonizing them and enhancing their phagocytosis; however, these antibodies can also cross-react with epitopes of PF4–heparin antigenic complexes, leading to their detection with commercially available anti-PF4 assays even in the absence of prior heparin exposure (Figure 1) [25]. Thus, PF4 represents an opsonin to defend against bacteria, and antibodies detected during HIT testing can be seen as a bacteria-targeted humoral immune response [25]. Evidence for this can be found in the rapid formation of IgG antibodies to PF4–heparin occurring soon after first heparin exposure, suggesting that IgM is pre-formed at the time heparin was given and that bacterial surface polyanions are the original offending antigen [26,27]. Furthermore, the ability of PF4 to bind with increasing avidity progressively truncated forms of LPS found on the surface of Gram-negative bacteria, as shown by the improved binding to phosphates on lipid A molecules devoid of the O antigen and the LPS core, typifies epitopes that mimic PF4–heparin complexes [28]. This provides evidence of a conserved anti-bacterial immune mechanism mediated by platelets’ PF4 [28].

Platelet presence is essential at the crossroads between inflammation and thrombosis. This relationship is indicated by results obtained from annexin knockout mice. In this model, reperfusion injuries show greater platelet adherence, and administration of annexin A1 directly upregulated thromboxane B and modulated phosphatidylserine expression on platelets, resulting in cerebral protection through both a reduction in platelet activation and enhanced phagocytosis by neutrophils [29]. Likewise, platelets, through their toll-like receptors (TLR) and adhesion molecules, sense their immediate microenvironment and function to recruit neutrophils and monocytes to infection sites while stimulating inflammatory cascades [30,31,32]. Platelets also modulate macrophage responses so that pro-inflammatory mediators release is reduced, thus rescuing mice from septic shock via a cyclooxygenase 1-dependent signaling pathway [33]. These effects, however, can be affected by the age of the donor since platelets from older subjects have been shown to both aggregate monocytes and worsen pro-inflammatory responses [34]. Collectively, these findings stress that the interplay between inflammation and thrombosis is highly complex and will require extensive investigation.

## 3. Immune-Associated Platelet Functions in Sepsis

Platelets are quantitatively the most abundant cellular blood element and represent a native surveillance system hunting for foreign molecules [35]. As a result, thrombocytopenia seen in sepsis may represent a process during which platelets that came into contact with foreign mediators/organisms are actively removed from circulation or are consumed due to tissue damage caused by the infection. For example, in viral infections such as dengue, decreases in platelet counts are observed soon after exposure to the virus, with thrombocytopenia becoming apparent as early as day 4 [36]. Specifically, the number of dengue viral copies inside platelets correlate with C3 and IgG binding, increased surface P-selectin expression indicative of platelet activation, and enhanced clot formation [36]. These infected platelets are in turn phagocytosed by monocytes, leading to their clearance from circulation [36].

During sepsis, epidermal growth factor receptor on platelets is central to their activation so that platelets can attract and augment macrophages’ immune function through the production of reactive oxygen species (ROS), while concurrently upregulating their pro-inflammatory macrophage M1 phenotype through increased expression of inducible nitric oxide synthase and CD64, resulting in bacteria clearance [34]. Similarly, platelet collagen receptor glycoprotein VI (GPVI) has been shown to be essential to host defense, as indicated by data from GPVI^−/−^ knockout mice, which have impaired platelet activation and platelet–leukocyte complex formation, leading to increased *Klebsiella pneumoniae* growth and infection [37]. This is of importance since GPVI is a member of the immunoglobulin receptor superfamily and the major receptor for collagen, a prime trigger for platelet activation [38]. Platelets also efficiently directly kill Gram-positive bacteria in an FcγRIIa-independent manner, as indicated by results using *Staphylococcus aureus*, and this process does not require PF4 opsonization but the release of bactericidal mediators [39]. This contrasts to results obtained with Gram-negative shiga-toxin-producing *Escherichia coli* O157:H7 infection, which reduced CD47 expression on platelets in a TLR-dependent manner, resulting in enhanced platelet phagocytosis by activated macrophages [40].

Platelets express Fc receptors for antigen recognition [41,42], as well as the pro-inflammatory mediators IL1-β and CD40 ligand (L) [43,44]. The latter is important since the adaptive immune response requires CD40 and soluble CD40L, both of which are actively secreted by activated platelets in response to microbial infection [24,45,46]. Furthermore, platelets readily activate proteins of the complement pathway, which further activate and recruit additional platelets to the site of infection [47]. Concomitant interaction of platelets with hepatic Kupffer cells represents an additional mechanism by which microbial-platelet clearance occurs [48]. Therefore, the phagocytic capabilities of platelets constitute an evolutionarily preserved immune mechanism since thrombocytes of non-mammalian lower vertebrates readily internalize bacteria, which are then quickly killed through phagolysosomal fusion [49,50].

Platelet–leukocyte contact also depends on the expression of cell adhesion molecules such as P-selectin and receptors that bridge endothelial cell communication with leukocytes [51]. In regard to how activated platelets stimulate monocytes via cytokines such as TGF-β, it has been shown that CD16 expression on monocytes is induced by platelets, causing them to develop into intermediate CD14^+^CD16^+^ cells that subsequently differentiate into M2 macrophages capable of antibody-dependent cellular phagocytosis [52]. Platelets are also necessary for the formation of neutrophil extracellular traps (NETs), which are partly triggered by platelet–leukocyte interactions and the release of proinflammatory mediators from platelets, thus indirectly trapping bacteria, viruses, and fungi, diminishing their propagation [53]. This occurs through the binding of platelet TLR4 to bacterial LPS, leading to platelet–neutrophil binding, which induces NET formation [54]. This has been confirmed in human infections with either Gram-negative or Gram-positive bacterial infections [55]. Platelets’ phosphatidylinositol 3-kinase catalytic subunit p110β has also been shown to be important for platelet–neutrophil/monocyte binding that, when deficient or inhibited, results in bacterial dissemination [56]. These results are important since p110β has also been shown to be involved in tumorigenesis and inflammation [57,58], providing evidence that these processes are closely associated under physiological conditions.

Platelet interactions with bacteria that either induce phagocytosis or activate platelets to initiate an immune response can be subdivided into three major categories: direct interaction, indirect, and interactions mediated by secreted mediators. These subsequently lead to three distinct mechanisms: adhesion, phagocytosis, and activation [59,60]. An example of direct platelet activation by bacteria is the binding of the Hs antigen expressed by strains of Streptococci to platelet’s GP1b [61]. An example of indirect interaction is the binding of bacterial protein A to plasma’s vWF followed by binding of the complex to Gp1b [62]. An example of the third category is when bacteria secrete immunogens that initiate a thrombin-like cascade through the activation of protease-activated receptor-1 [63]. This activation leads to subsequent release of bactericidal mediators present in platelet granules, in addition to the formation of platelet-bacteria thrombi as seen in sepsis and disseminated intravascular coagulation [59].

By now, it should not come as a surprise that the role of platelets as bonafide immune cells is coming into focus since the cellular ancestor of platelets and leukocytes, the thrombocyte, is pivotal to the immune systems of fish and birds [24,58]. It is with this knowledge that platelets’ role during infection should be seen as multifaceted and not driven by their hemostatic function. Their central place as part of an evolutionarily preserved immune response to defend the body from infectious threats should expand our understanding of additional effects when they are used during transfusions, especially when patients are treated for their thrombocytopenia.

## 4. Clinical Implications

Thrombocytopenia in sepsis holds significant clinical importance, serving not just as a laboratory finding but as a vital prognostic indicator that can profoundly impact patient management and outcomes [64,65,66]. Studies have shown that septic patients with thrombocytopenia face a higher risk of adverse outcomes, including bleeding complications, prolonged intensive care unit (ICU) stays, and overall increased mortality, compared to subjects with normal platelet counts [67]. For instance, a retrospective study involving 7981 patients revealed that a ≤10% reduction in platelet counts was associated with significantly lower 30-day mortality and shorter ICU stays compared to those with >10% reduction [13]. This study highlighted that thrombocytopenia in septic shock is associated with higher mortality, with case-fatality rates proportionally increasing with greater severity of thrombocytopenia.

**Figure 2:**
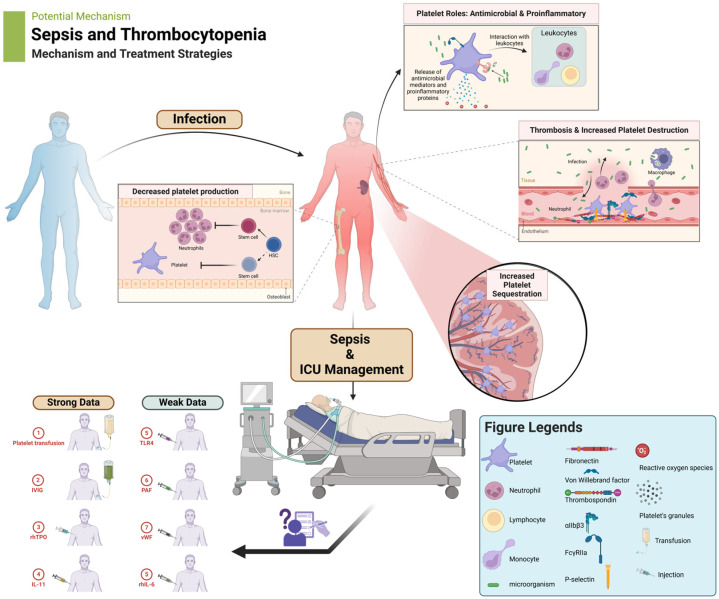
This figure illustrates the mechanisms of sepsis-related thrombocytopenia, including decreased platelet production, increased destruction, and sequestration. It also outlines treatment strategies such as platelet transfusion; IVIG; rhTPO; IL-11; and targeting of the TLR4, PAF, and vWF pathways. Abbreviations: HSC (hematopoietic stem cell), IVIG (intravenous immunoglobulin), rhTPO (recombinant human thrombopoietin), IL-11 (interleukin-11), TLR4 (Toll-like receptor 4), PAF (platelet-activating factor), vWF (von Willebrand factor), FcγRIIa (Fc gamma receptor IIa), aIIbβ3 (integrin alpha-IIb beta-3), and rhIL-6 (recombinant human interleukin-6). Figures were generated with BioRender.com.

The degree of thrombocytopenia in sepsis also directly correlates with the presence and worsening of multi-organ dysfunction [65]. This correlation implies that the correction of thrombocytopenia as well as targeted antimicrobial coverage has the potential to be a therapeutic intervention that can more significantly improve prognosis and influence outcomes during sepsis (Figure 2). Additional factors contributing to the development of thrombocytopenia in sepsis include higher sequential organ failure assessment scores, low PaO2/FiO2 ratios, and requirement for high vasopressor doses [64]. The poor response to platelet transfusions in sepsis, as indicated by low corrected count increments [68], is influenced by increased platelet consumption or destruction, fever, and conditions like splenomegaly and disseminated intravascular coagulation (DIC) that may develop [68,69]. As a result, a clear understanding of these factors is crucial for optimizing transfusion strategies and improving patient outcomes. However, despite the diminished platelet transfusion response, some studies have suggested that outcomes such as mortality and red blood cell (RBC) transfusion needs do not differ between patients with good and poor platelet increments [69].

Even though the management of thrombocytopenia in sepsis remains controversial due to conflicting evidence for and against platelet transfusions, there is an understanding among clinicians and researchers that this needs to be addressed to improve outcomes [70,71]. Further research is fundamental to establish a unified approach to manage SAT that takes its multifactorial nature under consideration. Thus, emerging therapies in treating sepsis, such as the use of intravenous immunoglobulins (IVIGs) and even administration of recombinant human thrombopoietin (rhTPO), show promise in enhancing platelet production and possibly reducing their destruction, offering potential new strategies for managing thrombocytopenia in such setting [72,73].

## 5. Management of Thrombocytopenia

### 5.1. Platelet Transfusions

The normal platelet count range in adults is 150–400 × 10⁹/L, with females generally exhibiting higher counts than males [74]. Thrombocytopenia is defined as a platelet count below 150 × 10^9^/L [75], and its severity guides clinical decision-making. Mild thrombocytopenia (100–150 × 10⁹/L) warrants close monitoring for progression and worsening clinical picture. Moderate thrombocytopenia (50–100 × 10⁹/L) often requires interventions to address underlying causes, as in sepsis, and platelet transfusion should be considered if the bleeding risk is high. Severe thrombocytopenia (<50 × 10^9^/L) necessitates urgent intervention, including platelet transfusion and aggressive treatment of the underlying condition [76]. In severe sepsis, approximately 40% of patients experience platelet counts below 80 × 10^9^/L, with decreasing platelet levels, as mentioned earlier, correlating with infection severity [74]. According to the Surviving Sepsis Campaign guidelines, platelet transfusion is recommended when counts drop below 10 × 10^9^/L, even in the absence of bleeding [77]. For patients at risk of significant bleeding or undergoing invasive procedures, transfusion is advised if platelet counts are below 20 × 10^9^/L [70], though these data remain controversial. These thresholds provide clinicians with a framework to assess the urgency of intervention and guide transfusion decisions in critically ill patients. Along these lines, the Association for the Advancement of Blood and Biotherapies (AABB) has provided recommendations for platelet transfusion thresholds for hospitalized patients, specifically, transfusion for platelet counts ≤10 × 10^9^/L to prevent spontaneous bleeding, prophylactic transfusion for counts <20 × 10^9^/L for central venous catheter placement, and platelet transfusions when patient is <50 × 10^9^/L during neurological surgery, among others [76]. However, consensus thresholds that are specific for SAT are lacking.

In terms of logistics, platelet units are one of the most expensive blood components in a blood bank inventory. These precious units must contain 3 × 10^11^ platelets per unit as required by government agencies such as the United States Food and Drug Administration. Units below this count are considered suboptimal and are mostly not released for transfusion by blood suppliers to hospitals. Platelet units are obtained from adults with normal platelet counts using either apheresis procedures or derived from whole blood donations. Although platelets from other sources such as cord blood may represent alternatives to adult donors, the counts in these cords are normally in the range of 210–265 × 10^9^/L, but since the amount of blood derived from a cord is 60–90 mL, it would require many cords to make a transfusable platelet dose, with many platelets being lost during the processing to remove RBCs [78,79]. Similarly, the use of human leucocyte antigen (HLA)-matched platelets for a specific recipient, due to their rarity, cost associated per unit (four times the cost of a regular unit), and possibly more limited donor pool for a given unit needed per patient, would make their use for routine transfusion prohibitive. This is the reason for accrediting organizations such as AABB and the published literature recommend that they should be reserved for patients who are shown to be refractory to routine platelet transfusions [80]. These are some of the factors to be considered when using platelet transfusions.

The majority of clinical trials looking at platelet transfusions in the setting of an infection have focused on hematology patients, creating a significant data gap for septic populations [81]. In this setting, prophylactic platelet transfusion in patients with severe thrombocytopenia (10–50 × 10^9^/L) showed reduced bleeding incidents in transfused patients, even among those in ICUs [81]. Retrospective studies, however, have found conflicting results in which higher platelet transfusion thresholds (20–50 × 10^9^/L) compared to lower thresholds (<20 × 10^9^/L) were associated with reduced mortality [70], while others saw increased mortality, fewer ICU-free days, and similar RBC transfusion rates with such thresholds [71]. A closer look at the impact of platelet transfusions on mortality in sepsis patients with severe thrombocytopenia (≤50 × 10^9^/L) found that platelet transfusions in this group were associated with higher in-hospital mortality rates but no effect in either 90-day mortality or ICU length of stay [82]. Thus, platelet transfusions in sepsis patients present a complex picture in regard to overall therapeutic benefit. When adverse outcomes to transfusions occur, they are made worse by the frailty of recipients, severity of underlying clinical condition, possible complications such as volume overload compounded by excessive transfusions and infusion of fluids, transfusion reactions, increased risk of thrombosis, and immune dysfunction [71,83,84,85]. Moreover, platelet transfusions can contribute to microvascular dysfunction by exacerbating the activation of the coagulation cascade while simultaneously inhibiting anticoagulation and fibrinolysis [71,84,86].

Despite these stated risks, platelet transfusions may offer benefits to still-to-be-defined groups of patients with sepsis by improving endothelial barrier function, reducing inflammation through their immunomodulatory effects, and promoting tissue repair [87,88]. As a result, having a clear understanding of the etiology of thrombocytopenia—whether due to hypersplenism, bone marrow suppression, medication-induced (HIT), or secondary to dilutional effects—is essential for optimizing clinical management. Under specific circumstances, strategies such as leukoreduction, platelet additive solutions, or HLA- and human platelet alloantigens-matched transfusions could improve count increments, but their benefit in sepsis patients still requires formal investigation [86,89].

### 5.2. Intravenous Immunoglobulins (IVIG)

The administration of IVIG as a potential therapeutic approach for thrombocytopenia, particularly when immune-mediated platelet destruction is suspected, may not be without merit [90]. IVIG modulates the immune response by disrupting autoantibody-mediated platelet clearance [91], making it a plausible treatment option in SAT [92,93], especially when mechanisms mirror those encountered in immune thrombocytopenia [94,95]. It is believed that IVIG is able to do this through its anti-idiotypic effect over circulating antibodies. In this case, elevated levels of platelet-associated IgG observed in septic thrombocytopenia further support its use, and future research should establish its role as a therapeutic approach [94].

The results from a randomized blinded clinical trial found that IVIG significantly increased platelet counts in patients with septic thrombocytopenia, increasing counts by 411% in the IVIG group versus 261% in the placebo group by day 9 [94]. Additionally, a report of a septic COVID-19 patient with severe immune thrombocytopenia showed rapid platelet count recovery and improved oxygenation after combined IVIG with corticosteroid therapy without occurrence of adverse events [96]. Along these lines, reports of pregnant women with thrombocytopenia and signs of infection showing a >60% improvement in counts after receiving IVIG with low-dose rhTPO highlights its therapeutic potential in specific patient populations when using combined therapy [93].

In contrast, IVIG therapy in severe fever with thrombocytopenia syndrome (SFTS) has been associated with higher fatality rates and significant adverse events, such as worsening viral loads and suppressed immune cell counts [97]. These findings, derived from a retrospective study, warrant cautious interpretation due to its methodological limitations, including single-center design, SFTS patient selection, and lack of randomization. Interestingly, IgM-enriched IVIG significantly reduced mortality, hospital stays, and APACHE II scores in sepsis patients, showing greater efficacy compared to standard IVIG [98]. Even when higher doses of IVIG were used, therapeutic benefit has been reported through the rapid inhibition of platelet activation, lending support to a broader role in managing thrombocytopenia occurring secondary to immune dysregulation [99,100].

Altogether, IVIG shows promise in rapidly increasing platelet counts and improving outcomes in selected patients with SAT [94,96,98]. Its use may be particularly beneficial for those at high risk of bleeding or undergoing invasive procedures. However, careful patient selection and further studies are needed to establish its true efficacy and safety either as a standalone therapy or in combination with other treatments.

### 5.3. Recombinant Human Thrombopoietin (rhTPO)

This recombinant growth factor effectively increases platelet counts with minimal side effects by promoting differentiation and maturation of bone marrow stem cells into megakaryocytes, thereby enhancing platelet production without altering their morphology or function [72,101,102,103]. These benefits have been well demonstrated during chemotherapy-induced, immune-mediated, and sepsis-associated thrombocytopenia, resulting in overall lower transfusion dependency [101,104,105,106]. A meta-analysis of ten randomized controlled trials (RCTs) involving 681 patients with SAT showed that rhTPO significantly increased platelet counts, decreased 28-day mortality, reduced number of platelet units transfused, reduced transfusion volumes, and shortened ICU stays compared to conventional antibiotic therapy alone or in combination with IVIG [72]. Furthermore, retrospective data of 213 patients lend support to rhTPO use, showing greater platelet count increases, specially in patients with initial counts ≤30 × 10^9^/L and APACHE II scores > 15 [103]. Even though this growth factor is costly, the reduction in ICU stays may provide a strong incentive for its use even when 28-day mortality may remained unchanged [103].

### 5.4. Recombinant Human IL-11

Interleukin-11 (IL-11), a cytokine interacting with hematopoietic and non-hematopoietic cells, has demonstrated therapeutic potential in systemic inflammatory conditions including sepsis [107,108]. Specifically, its strong thrombopoietic effect favors its use as potential therapy for SAT [108,109]. A study of 105 patients found that treatment with IL-11 significantly improved platelet counts and reduced IL-6 levels between days 3 and 14 [110]. The therapy also downregulated the sepsis marker procalcitonin and lowered the APACHE II score of patients, in a background of lower 28-day mortality rate compared to conventional therapy [110]. However, IL-11’s role in treating SAT remains mostly underexplored. Although preliminary studies suggest its efficacy in light of improved clinical outcomes, the data are still limited and RCTs are needed to validate its therapeutic potential in septic patients [108].

### 5.5. Recombinant Human IL-6

Elevated IL-6 levels, a hallmark of sepsis, correlate with disease severity and act as diagnostic and prognostic markers [111]. Notably, there is an inverse relationship between thrombocytopenia and cytokine activation in sepsis [112], prompting exploration of anti-IL-6 therapies to mitigate inflammation and restore platelet counts since this cytokine is likely a key driver of the cytokine storm in sepsis. Tocilizumab (TCZ), a recombinant humanized monoclonal antibody targeting the IL-6 receptor, has shown potential in managing sepsis, particularly in COVID-19 patients [113,114]. By inhibiting both membranous and soluble IL-6 receptors, TCZ suppresses the IL-6-mediated inflammatory cascade [115]. However, TCZ’s role is complex; while it may dampen cytokine-driven platelet consumption, reports of TCZ-induced thrombocytopenia raise concerns [113]. This physiologic duality underscores the need for further research into its safety and efficacy in septic patients, especially those with existing thrombocytopenia. With this in mind, the RESCUE trial aiming to further evaluate IL-6 inhibition on patients with inflammation and atherosclerotic disease via another monoclonal antibody ziltivekimab will provide needed evidence to establish if inhibition of this cytokine proves beneficial to patients [116].

### 5.6. TLR4 Inhibition

TLR4 is a key membrane-spanning protein in the innate immune system, recognizing pathogen-associated molecular patterns such as LPS from Gram-negative bacteria [117,118]. Expressed on various cell types, including platelets, TLR4 activation triggers intracellular signaling cascades, leading to NF-kB pathway activation that results in the release of pro-inflammatory cytokines. This process contributes to systemic inflammation and sepsis progression, positioning TLR4 as a potential therapeutic target for modulating sepsis-associated inflammation [31]. TAK-242, a TLR4 antagonist, has shown promise as adjunct therapy for thrombocytopenia in sepsis by mitigating LPS-induced systemic inflammation, thus reducing end-organ damage and slowing sepsis progression [119,120,121]. Its ability to specifically target inflammatory pathways underscores its potential to improve outcomes in septic patients with thrombocytopenia.

Thrombocytopenia and platelet dysfunction are associated with sepsis-induced organ failure and platelets’ mitochondrial ROS (mtROS) and autophagy. Since TLR4 is central during both of these processes, the use of TAK-242 has been shown to improve the function of lungs and kidneys in septic mice through the inhibition of platelet GPIIb/IIIa and a reduction in LPS-induced mtROS generation, resulting in reduced platelet activation [119]. Tissue damage is due to worsening injury partly driven by extensive platelet aggregation secondary to LPS exposure and TLR4 activation. Thus, TAK-242 administration decreases in LPS-mediated P-selectin expression, and intracellular ROS production improves tissue damage [122]. However, the extent of platelet activation appears to be dependent upon the type of LPS present, since marked differences have been reported upon exposure to different bacterial LPS in their capacity to aggregate platelets, trigger granule secretion and cytokine release, produce ROS, interact with leukocytes, and even form NET [123]. Platelet activation, nonetheless, is not limited to TLR4 since *Streptococcus pneumoniae* and Group B Streptococcus have been shown to activate platelets, induce CD62P expression and secretion, and activate GPIIb/IIIa via TLR2 [124,125]. LPS even enhances aggregation of washed platelets stimulated by thromboxane or GPVI collagen receptor agonists, effects that can be prevented by a TLR4 antagonist [126]. These LPS-mediated effects were associated with the phosphorylation of Akt, ERK1/2, and PLA2 in stimulated platelets; on the other hand, inhibitors of phosphorylation resulted in enhanced platelet function and lower ROS production [126]. These LPS-driven effects are also partly due to the activation of TLR4/NF-κB interaction, since inhibition of this pathway improves sepsis-induced tissue injury [127]. This is the case as well in endotoxin-mediated muscle damage occurring during infection, which TAK-242 reversed through action over the same pathway [128].

The activation of TLR4 on platelets by LPS also triggers pro-thrombotic and pro-coagulant responses, including platelet aggregation, ATP release, P-selectin expression, and platelet–neutrophil aggregate formation [129]. These mechanisms contribute to both inflammation, coagulopathy, and DIC seen in sepsis. TLR4 also interacts with CD14, damage-associated molecular patterns (e.g., HMGB1), and NETs, amplifying inflammatory and coagulation pathways that link thrombocytopenia and sepsis progression [120,121,129]. Despite mechanistic evidence supporting TLR4’s role in thrombocytopenia in sepsis, clinical trials investigating its therapeutic potential remain lacking. Future studies will be needed to translate these findings into effective clinical interventions.

### 5.7. Platelet-Activating Factor (PAF) Inhibition

PAF’s role in LPS-induced thrombocytopenia and neutropenia was shown by a study looking at 42 dogs treated with LPS, PAF, or saline, with some animals being pre-treated with TCV-309, a potent PAF antagonist. Thrombocytopenia and neutropenia occurred in all but the saline group, with TCV-309 significantly reducing LPS-induced thrombocytopenia, suggesting PAF as a mediator and potential therapeutic target for SAT [130]. PAF appears central to severe sepsis, where bacterial endotoxins trigger its production, leading to hypotension, organ damage, and thrombocytopenia [130]. The use of recombinant PAF-acetylhydrolase demonstrated reduced mortality in severe sepsis subjects, underscoring its therapeutic potential [131]. Moreover, PAF receptor (PAF-R) activation exacerbates inflammatory responses in infectious diseases, such as dengue fever, where it worsens thrombocytopenia and increases mortality [132]. Also, its role in inflammation related to HIV and SARS-CoV-2 further emphasizes its relevance across infectious conditions [132]. Thus, targeting PAF-R represents a promising approach for treating SAT. Clinical trials to study the safety and efficacy of PAF inhibitors in the setting of sepsis are justified.

### 5.8. Von Willebrand Factor (vWF)-Binding Function

Sepsis disrupts the delicate balance between vWF and the enzyme known as a disintegrin and metalloproteinase with thrombospondin type 1 motif 13 (ADAMTS13), leading to lower ADAMTS13 activity in a background of elevated vWF levels [133,134]. This imbalance results from increased vWF and removal of ADAMTS13 from circulation rather than intrinsic changes in ADAMTS13 itself, which when persisting even post-ICU, augments thrombotic risks [133]. Studies in *Staphylococcus aureus*-associated sepsis further emphasize this imbalance’s impact. In this research, high vWF and low ADAMTS13 activity correlate with severe illness in humans, while in mice, vWF deficiency improves survival and ADAMTS13 deficiency worsens it [134]. Similarly, GPIb-IX, which appears to be important for vWF-mediated platelet adhesion, emerges as a potential therapeutic target. In LPS-induced sepsis models, a dysfunctional GPIbα mutant or the synthetic vWF-binding inhibitor MPαC significantly reduced mortality, thrombosis, and platelet loss [135]. These findings position vWF-mediated adhesion as a target for development of therapies in SAT. This section is summarized in Table 1.

## 6. Biomarkers

Predicting severe thrombocytopenia in sepsis remains a challenge, but a combination of biomarkers and clinical parameters offers promising avenues for risk stratification. No single biomarker is universally accepted as a perfect predictor, but several have demonstrated diagnostic potential. These include platelet count and its trend (both initial count and rate of decline), mean platelet volume, platelet distribution width [74], immature/reticulated platelet fraction/counts [139,140], inflammatory markers (C-reactive protein, procalcitonin, and IL-6) [141], coagulation markers (D-dimer, fibrinogen, and antithrombin III [142,143], liver function markers (bilirubin and albumin) [144], endothelial dysfunction markers (vWF, Angiopoietin-2, and nitric oxide) [145], immune system markers (neutrophil-to-lymphocyte ratio), and organ dysfunction scores (Sequential Organ Failure Assessment, Acute Physiology and Chronic Health Evaluation II, Injury Severity Score, and Revised Trauma Score) [146]. Emerging research is also exploring genetic and molecular biomarkers like microRNAs and TLR signaling markers [147,148]. Clinical risk factors such as age, comorbidities (especially liver, kidney, or hematologic disorders) [149], and the source of infection (e.g., Gram-negative bacteria) also contribute to the overall risk assessment [150]. Thus, integrating these variables remains essential for a more accurate clinical prediction.

## 7. Future Research Directions

While the role of platelets in hemostasis is well established, their immune functions in sepsis remain incompletely understood. Future research shall focus on elucidating the precise molecular mechanisms by which platelets interact with pathogens, immune cells, and endothelial cells during sepsis. For example, the role of platelet-derived mediators such as PF4, TLR4, and CD40L in modulating immune responses and bacterial clearance warrants further investigation [151]. Additionally, the interplay between platelet activation, NET formation, and cytokine release in SAT needs to be explored in greater detail [53,54]. Furthermore, a better understanding will allow for the development of approaches that enhance positive effects while attempting to limit those that adversely affect septic patients (Table 1).

Identifying reliable biomarkers for predicting the development and severity of thrombocytopenia in sepsis could improve risk stratification and guide therapeutic interventions [152]. Future research should focus on validating biomarkers such as those mentioned above, in addition to immature (reticulated) platelet counts, any fluctuations in ADAMTS13 activity, vWF levels, and changes to cytokine profiles (e.g., IL-6 and IL-11) [153]. Additionally, the use of machine learning and artificial intelligence to integrate clinical, laboratory, and biomarker data for predicting outcomes in septic patients with thrombocytopenia should be explored [154].

The efficacy and safety of platelet transfusions in septic patients with thrombocytopenia remain controversial [70]. Future studies should aim to identify specific patient subgroups that benefit from platelet transfusions, such as those with severe thrombocytopenia or high bleeding risk. RCTs are needed to evaluate the impact of different transfusion thresholds (e.g., 10 × 10^9^/L vs. 20 × 10^9^/L) on clinical outcomes, including mortality, bleeding events, and ICU length of stay. Furthermore, the role of leuko-reduced, HLA-matched, or newly adopted pathogen-reduced platelet transfusions in reducing adverse effects and improving outcomes in sepsis should be investigated.

As mentioned earlier, emerging therapies such as rhTPO, IVIG, and TLR4 inhibitors show promise in managing SAT. Future research should focus on optimizing the dosing, timing, and combination of these therapies to enhance platelet production and reduce immune-mediated platelet destruction [73,98]. For instance, the potential synergistic effects of combining rhTPO with IVIG or TLR4 antagonists in septic patients with thrombocytopenia should be explored in preclinical and clinical studies [155]. Additionally, the role of PAF inhibitors and vWF-binding inhibitors in mitigating thrombocytopenia and inflammation in sepsis warrants closer scrutiny.

Aging and comorbidities such as diabetes, cancer, and chronic kidney disease can alter platelet function and immune responses, potentially influencing the clinical course of sepsis [156]. Future studies should investigate how these factors affect platelet activation, immune modulation, and response to therapies in septic patients. For example, the impact of age-related changes in platelet TLR4 signaling or the role of comorbidities in exacerbating sepsis-induced thrombocytopenia should be examined. Furthermore, given the central role of platelets in immune defense, future research ought to explore the development of platelet-directed immunotherapies for sepsis. For instance, engineered platelets with enhanced antimicrobial or anti-inflammatory properties could be investigated as a novel therapeutic approach [157]. Additionally, the use of platelet-derived exosomes or microvesicles as carriers for targeted drug delivery to sites of infection or inflammation in sepsis represents an interesting area of investigation.

Survivors of sepsis often experience long-term sequelae, including persistent thrombocytopenia, immune dysfunction, and increased risk of thrombosis [158]. Future studies will be needed to determine the long-term impact of SAT on platelet function, immune recovery, and thrombotic risk in sepsis survivors. Understanding these outcomes could inform the development of strategies to mitigate long-term complications and improve quality of life in this patient population. By addressing these research gaps, we can deepen our understanding of the multifaceted role of platelets in sepsis and develop more effective strategies for managing thrombocytopenia and improving patient outcomes.

## 8. Discussion

Thrombocytopenia in sepsis is a complex clinical complication with significant implications for patient outcomes. It presents frequently in sepsis, affecting nearly half of septic patients and serving as a critical and negative prognostic marker [158,159]. While traditionally viewed as primarily one of the most important hemostatic mediators [160], platelets play a crucial role in the immune response to infection [161]. Thus, thrombocytopenia has profound immune repercussions in patients battling infections. By directly interacting with pathogens, modulating inflammatory responses, and promoting immune cell function, platelets’ roles in both innate and adaptive immunity cannot be denied [19].

The pathogenetic mechanisms seen in sepsis can involve decreased platelet production due to bone marrow suppression, increased destruction through DIC, and platelet sequestration in the spleen and other organs [115,158,159]. The totality of findings presented here makes the complex interplay of pathophysiological mechanisms and the clinical impact over the possible management strategies needed for thrombocytopenia in sepsis evidently clear [115]. The clinical implications are profound; thrombocytopenia correlates with higher risks of bleeding, prolonged ICU stays, and increased mortality [158]. Specially, studies consistently show that even a modest reduction in platelet count is associated with worse outcomes, emphasizing the importance of frequent monitoring and timely intervention [13]. Therefore, the cumulative data underscore the complexity of thrombocytopenia in sepsis and the necessity for targeted management strategies.

A critical aspect of managing septic patients is the consideration of platelet transfusions. While platelet transfusions are often employed to treat thrombocytopenia, their efficacy in sepsis has remained controversial [162,163]. It is the routine use of platelet transfusions, despite limited efficacy data, which could limit the adoption of novel therapies by obscuring the effect of such therapies given contemporarily to the transfusion. Advancing platelet-based treatments for sepsis require deeper research, including biomarker-driven approaches, and likely combination strategies. Thus, multidisciplinary collaboration and innovative trials are key to translating their usefulness into clinical practice. As a result, the timing of transfusion, number of platelet units given, and underlying disease severity could influence outcomes. While some studies have suggested benefits in reducing bleeding and improving survival, particularly with higher transfusion thresholds [70,163], others report no improvements in outcomes, instead suggesting increased mortality among recipients, possibly due to the severity of illness and complications such as transfusion-related infections, immune dysfunction, and the immunomodulatory effects mediated by platelets themselves [71].

Emerging therapies such as rhTPO and immunomodulatory agents offer potential for enhancing platelet production and reducing destruction [163]. As pointed out earlier in the text, IVIG has shown promise in this regard, particularly in cases where immune-mediated platelet destruction is suspected. RCTs and case studies highlighting IVIG’s ability to significantly increase platelet counts and improve clinical outcomes in SAT are encouraging [93,94]. However, the effectiveness of IVIG is not universal. For instance, in SFTS, IVIG treatment was associated with increased mortality and adverse immunological effects, highlighting the need for careful patient selection and monitoring after its use [97]. In addition to transfusions and IVIG, adjunctive therapies targeting specific pathways involved in platelet production and destruction are potential therapies that can help patients. These include agents that are immunomodulatory in nature, inhibit platelet receptor interactions, and address underlying conditions such as hypersplenism and bone marrow suppression [130,131,133]. The use of high-dose IVIG in conditions like HIT illustrates the potential for targeted therapies to mitigate platelet activation and limit platelet consumption [99].

Without a doubt, developing and translating novel platelet-based therapies for sepsis into clinical practice presents significant challenges. The complexity of platelet biology complicates targeted treatments, as platelets play dual roles in hemostasis and immunity [164]. Therapies addressing one function may inadvertently disrupt another, leading to unintended consequences such as increased bleeding or impaired immune defense. Additionally, platelet responses vary based on sepsis stage, pathogen type, and patient-specific factors, making a one-size-fits-all approach difficult [165]. A major obstacle is the lack of clear biomarkers to identify patients who would benefit most from platelet-based therapies, as platelet counts fluctuate and sepsis progressively evolves [166]. Preclinical models could also hinder progress, as animal studies often fail to fully replicate human pathophysiology, limiting translational potential [167]. In addition, regulatory concerns regarding thrombosis, bleeding risks, and immune modulation from these approaches require extensive safety data collection that include the determination of long-term effects [76]. Economic and logistical barriers, including high development costs, complex manufacturing processes, and limited pharmaceutical incentives, further slow progress. Finally, clinical trials face additional hurdles due to the heterogeneity of sepsis patients, lack of standardized endpoints, and ethical challenges in critically ill populations [168]. These difficulties will need to be addressed in the future to establish the best evidence-based therapeutic approaches.

## 9. Conclusions

In conclusion, thrombocytopenia in sepsis is a critical and complex condition with significant implications for patient management and outcomes. A comprehensive understanding of its pathophysiology and clinical impact, coupled with evidence-based management strategies, is essential for improving patient care. Future studies should aim to clarify the role of various treatment modalities, including platelet transfusions and adjunctive therapies as those presented here, in optimizing outcomes for septic patients with thrombocytopenia. Thus, the management of thrombocytopenia in sepsis likely requires a tailored approach that considers individual patient factors and the underlying pathophysiology of a patient’s presentation. Any research looking at this patient population should account for as many of these variables as possible and recruit patients accordingly so that the results can be extrapolated to address the diverse nature of thrombocytopenia in sepsis. Future research ought to include robust RCTs that establish the benefits of the approaches mentioned in this review. The addition of platelet function tests and advanced diagnostic tools could further refine treatment strategies, ensuring that interventions are timely, effective, and safe. Nevertheless, the data are clear that the armamentarium needed to treat thrombocytopenia in the setting of an infection is likely to grow in the coming years.

## Figures and Tables

**Table 1:** Summary of key findings, mechanisms, clinical relevance, and future directions in sepsis-associated thrombocytopenia (SAT).

Section	Key Findings	Mechanisms	Clinical Relevance	Future Directions
Platelet transfusions [71,76,82,83,84,85,87,88]	They are commonly used but have mixed outcomes in sepsis. Higher transfusion thresholds may reduce mortality, but risks include volume overload, thrombosis, and immune dysfunction.	They may exacerbate inflammation and coagulation. Platelet activation and microvascular dysfunction contribute to adverse effects.	Platelet transfusions are a mainstay for severe thrombocytopenia but require careful patient selection and monitoring to avoid complications.	Conduct RCTs to define optimal transfusion strategies and identify patient subgroups that benefit most.
Intravenous immunoglobulins (IVIG) [90,91,92,93,94,95,96,98,99,100]	IVIG boosts platelet counts in sepsis by modulating immune responses, potentially reducing immune-mediated destruction and improving outcomes.	IVIG inhibits autoantibody-mediated platelet clearance and modulates Fc receptor interactions. It reduces inflammation and enhances platelet survival.	IVIG is effective in immune-mediated thrombocytopenia and may benefit septic patients with similar mechanisms.	Evaluate IVIG in larger randomized trials for SAT and explore combination therapies.
Recombinant human thrombopoietin (rhTPO) [72,101,102,103,104,105,106]	rhTPO enhances platelet production and reduces transfusion dependency in sepsis. It improves platelet counts and reduces ICU stays, but its cost-effectiveness needs further evaluation.	rhTPO stimulates megakaryocyte differentiation and platelet production in the bone marrow. It does not alter platelet function or morphology.	rhTPO is a promising therapy for SAT, particularly in patients with severe thrombocytopenia and high disease severity.	Investigate cost-effectiveness and long-term outcomes of rhTPO in sepsis. Explore its use in combination with other therapies.
Recombinant human IL-11 [107,108,109,110]	IL-11 has thrombopoietic effects and improves platelet counts in sepsis. It reduces IL-6 levels and sepsis markers, but its role in SAT requires further investigation.	IL-11 stimulates megakaryopoiesis and platelet production. It also modulates inflammatory responses by reducing pro-inflammatory cytokines.	IL-11 shows potential in improving platelet counts and reducing inflammation in sepsis, but evidence is limited.	Conduct RCTs to validate IL-11’s efficacy and safety in SAT.
Recombinant human IL-6 [111,112,115,116,136,137]	IL-6 inhibition (e.g., tocilizumab) shows potential in managing sepsis and thrombocytopenia, but its dual role in platelet consumption and inflammation complicates its use.	IL-6 drives cytokine storms and platelet consumption in sepsis. Tocilizumab inhibits IL-6 receptors, reducing inflammation but may also cause thrombocytopenia.	IL-6 inhibition is a promising strategy for severe sepsis, but its effects on platelets require careful monitoring.	Further research into IL-6 inhibition’s safety and efficacy in sepsis, particularly in patients with thrombocytopenia.
TLR4 inhibition [117,118,120,121,129,138]	TLR4 activation contributes to sepsis progression and thrombocytopenia. TLR4 inhibitors (e.g., TAK-242) show promise in reducing inflammation and improving outcomes in sepsis.	TLR4 recognizes LPS and triggers NF-κB signaling, leading to pro-inflammatory cytokine release and platelet activation. TLR4 inhibition reduces inflammation and platelet consumption.	TLR4 inhibitors may improve outcomes in sepsis by reducing inflammation and thrombocytopenia.	Conduct clinical trials to evaluate TLR4 inhibitors’ efficacy in sepsis and their impact on thrombocytopenia.
PAF inhibition [130,131,132]	PAF mediates thrombocytopenia and inflammation in sepsis. PAF inhibitors (e.g., TCV-309) reduce thrombocytopenia and improve outcomes in animal models.	PAF activates inflammatory and pro-thrombotic pathways, contributing to platelet consumption and organ damage. PAF inhibition reduces these effects.	PAF inhibitors show therapeutic potential in sepsis, particularly for reducing thrombocytopenia and inflammation.	Investigate PAF inhibitors in clinical trials for sepsis, and explore their use in combination with other therapies.
vWF-binding [133,134,135]	Imbalance between vWF and ADAMTS13 in sepsis contributes to thrombosis and thrombocytopenia. Targeting vWF-mediated adhesion (e.g., GPIb-IX inhibitors) shows therapeutic potential.	Elevated vWF and reduced ADAMTS13 activity promote platelet adhesion and microthrombosis. Inhibiting vWF-GPIb interactions reduces platelet consumption and thrombosis.	Targeting vWF-mediated adhesion may reduce thrombocytopenia and thrombosis in sepsis.	Develop and test vWF inhibitors in clinical trials for sepsis-associated thrombocytopenia.

In each of the sections, the numbers in brackets represent the references in the text providing data for a given area.

## Data Availability

All relevant data have been provided in the text of this review.
